# High-performance spinel-rich Li_1.5_MnTiO_4+*δ*_ ultralong nanofibers as cathode materials for Li-ion batteries

**DOI:** 10.1038/srep45579

**Published:** 2017-03-31

**Authors:** Ngoc Hung Vu, Paulraj Arunkumar, Won Bin Im

**Affiliations:** 1 School of Materials Science and Engineering and Optoelectronics Convergence Research Center, Chonnam National University, 77 Yongbong-ro, Buk-gu, Gwangju 61186, Republic of Korea

## Abstract

Recently, composite materials based on Li-Mn-Ti-O system were developed to target low cost and environmentally benign cathodes for Li-ion batteries. The spinel-layered Li_1.5_MnTiO_4+*δ*_ bulk particles showed excellent cycle stability but poor rate performance. To address this drawback, ultralong nanofibers of a Li_1.5_MnTiO_4+*δ*_ spinel-layered heterostructure were synthesized by electrospinning. Uniform nanofibers with diameters of about 80 nm were formed of tiny octahedral particles wrapped together into 30 μm long fibers. The Li_1.5_MnTiO_4+*δ*_ nanofibers exhibited an improved rate capability compared to both Li_1.5_MnTiO_4+*δ*_ nanoparticles and bulk particles. The uniform one-dimensional nanostructure of the composite cathode exhibited enhanced capacities of 235 and 170 mAh g^−1^ at C/5 and 1 C rates, respectively. Its unique structure provided a large effective contact area for Li^+^ diffusion, and low charge transfer resistance. Moreover, the layered phase contributed to its capacity in over 3 V region, which increased specific energy (726 Wh kg^−1^) compared to the bulk particles (534 Wh kg^−1^).

Li-ion batteries (LIBs) have attracted enormous attention owing to their characteristic high energy densities and longer cycle life compared to other battery technologies^1^. Among commercial LIBs, spinel LiMn_2_O_4_ is considered to be the most promising cathode because of the abundance of manganese resources, their low cost, eco-friendliness, high theoretical capacities, and ease of synthesis, especially compared to LiCoO_2_, LiNiO_2_, and LiFePO_4_^2^. Spinel LiMn_2_O_4_ has a 3-D pathway for Li transport, which results in a high rate performance for LIBs. However, this material has some drawbacks, such as suffering from capacity fading during cycling caused by Jahn-Teller distortions and Mn dissolution^3^; these problems are especially severe during operation in a wide voltage range (e.g., 2.0–4.8 V)^4^,^5^. Overcoming these issues while preserving the advantages of spinel LiMn_2_O_4_ is challenging. Ti is the second most abundant transition metal in the Earth’s crust, benign to the environment, and forms stronger bonds with O than does Mn (662 kJ mol^−1^ and 402 kJ mol^−1^, respectively). Therefore, the substitution of Mn by Ti can improve the stability of the spinel framework without sacrificing the advantages of LiMn_2_O_4_^6^,^7^.

However, the substitution of Mn by Ti also leads to a decrease in the capacity of the cathode^8^. The specific capacity of cathode can be improved by the inclusion of excess Li, which results in a net increase in the available quantity of Li for intercalation^9^,^10^,^11^. Qien *et al*.^9^, reported that the addition of excess Li plays a significant role in tuning the Mn^3+^ content, phase composition, and purity of spinel cathodes. In addition, the excess Li results in an enhanced cycling and improved rate performance of the spinel cathodes. Hence, the addition of excess Li is a potential approach to achieve a superior electrochemical performance in spinel cathodes. We have previously reported a new material, Li_1+*z*_MnTiO_4+*δ*_ (where *z* = 0, 0.5 and 1.0, and 0 ≤ *δ* < 0.67), that has an improved capacity and cycling stability compared to current materials with cathode applications^12^. Among cathode materials containing excess Li, Li_1.5_MnTiO_4+*δ*_ shows the highest capacity. However, synthesis of Li_1.5_MnTiO_4+*δ*_ by solid-state reaction results in non-uniform, large particles, which limited the contact area with electrolyte, leading to a low capacity and poor C-rate performance. To address the low capacity and rate performance of these cathodes, a unique nanostructure with special morphology is crucial^13^,^14^,^15^.

One way to improve the capacity, as well as rate performance of the cathode material, is to exploit nanomaterials that have high surface areas, in particular, one-dimentional (1-D) nanomaterials^11^. However, it is very difficult to grow 1-D spinel Li_1.5_MnTiO_4+*δ*_ owing to its cubic crystal structure. Thus, it is challenging to synthesize ultralong nanofibers of cubic spinel Li_1.5_MnTiO_4+*δ*_, and few papers have reported the preparation of ultralong spinel nanofibers^16^,^17^. In contrast, electrospinning is a facile method for the synthesis of 1-D nanomaterials^18^. Electrospinning is a simple process that does not require a vacuum. Instead, a high voltage power supply, a spinneret containing a polymer solution, and a collector are required. This technique is widely used to fabricate nanofibers, nano/meso composites, core-shell materials, and hollow configurations^18^,^19^. Zhou *et al*.^16^, reported the synthesis of ultralong LiMn_2_O_4_ nanofibers by a combination of electrospinning and sol-gel techniques. The resulting cathode material had a porous “network-like” morphology with a nanodomain diameter (~170 nm) and microdomain length (~20 μm) with a pure spinel structure. Min *et al*.^20^, used electrospinning to synthesize Li_1.2_Ni_0.17_Co_0.17_Mn_0.5_O_2_ nanofibers that exhibited excellent rate capability when compared to co-precipitated Li_1.2_Ni_0.17_Co_0.17_Mn_0.5_O_2_ particles. Moreover, if one dimension of the nanocrystallites is up to a few hundred micrometers or even the millimeter scale, such as ultralong nanowires or nanobelts, self-aggregation of the nanomaterials can be effectively prevented. Therefore, to some extent, ultralong nanofibers are one of the most favorable structures for use as cathode/anode materials for high-performance LIBs^21^,^22^.

In this study, we successfully synthesized ultralong Li_1.5_MnTiO_4+*δ*_ nanofibers with lengths of 30 μm and diameters of 80 nm that had a spinel-layered heterostructures; this was achieved by optimizing concentration of precursor solution and adjusting the electrospinning conditions. The heterostructure of the nanofiber enables facile electron transport along the 1-D geometry and reduces the Li-ion diffusion length, resulting in an enhanced discharge capacity and excellent C-rate performance. The physical, chemical, and electrochemical properties of the Li_1.5_MnTiO_4+δ_ nanofibers were investigated by X-ray diffraction (XRD), field emission scanning electron microscopy (FE-SEM), high-resolution transmission electron microscopy (HRTEM), cyclic voltammetry (CV), electrochemical impedance spectroscopy (EIS), and galvanostatic cycling.

## Experimental

### Fabrication of the Li_1.5_MnTiO_4+*δ*_ nanofibers

The solution for electrospinning was prepared from polyvinylpyrrolidone (PVP, Sigma-Aldrich, *M*_w_ = 1300000), *N,N*-dimethylformamide (DMF, Sigma-Aldrich), acetic acid (Sigma-Aldrich), lithium acetate dihydrate (LiCH_3_COO·2H_2_O, Sigma-Aldrich), manganese acetate (Mn(CH_3_COO)_2_·4H_2_O), and titanium (IV) isopropoxide (Sigma-Aldrich). First, stoichiometric amounts of Mn(CH_3_COO)_2_·4H_2_O (0.005 mol), LiCH_3_COO·2H_2_O (0.0075 mol) were dissolved in DMF (15 mL). Then, titanium (IV) isopropoxide (0.005 mol) was dissolved in acetic acid (3 mL) before the addition of DMF (5 mL). The titanium (IV) isopropoxide solution was then mixed with the previous solution containing Mn and Li salts. The color of the mixture immediately became a clear dark yellow. After stirring for 15 min, PVP was added to the mixture. To control the diameter of the electrospun fibers, various concentrations of PVP were investigated. After optimization, we found that a concentration of 0.1 g/mL was optimal for the density and the size of nanofiber (Supplementary Figure S1). Polyvinyl acetate (PVA) was also tested, but because of the low degree of bonding with titanium (IV) isopropoxide^19^,^20^, the surfaces of nanofibers after firing were not smooth and the nanofibers were shorter (Supplementary Figure S2). Before electrospinning, the polymer mixture was stirred vigorously at room temperature for 24 h. A high voltage power supply (eS-robot^®^) was used to provide voltages of 15–20 kV for electrospinning. Fibers were collected on an aluminum foil as a mat. Following optimization, the nanofibers were calcined at 600 °C for 12 h in air to eliminate any organic residues. The firing temperature was chosen based on optimizations carried out in our previous publications^20^. The Li_1.5_MnTiO_4+*δ*_ nanofibers was denoted LMTO-NF.

### Preparation of Li_1.5_MnTiO_4+*δ*_ nanoparticles and bulk particles

The nanoparticles were prepared by sol-gel based Pechini method. Typically, LiCH_3_COO·2H_2_O (0.0075 mol), Mn(CH_3_COO)_2_·4H_2_O (0.005 mol), and citric acid (0.02 mol) were dissolved in a mixed solvent (15 mL, 2:1 v/v distilled H_2_O and ethylene glycol) while stirring to form a clear solution. Then, titanium (IV) isopropoxide (0.005 mol) was added dropwise to the above solution with stirring. The mixture was stirred vigorously at room temperature for 4 h and then heated to 140 °C to induce gelation. The obtained xerogel was further dried under vacuum at 120 °C for 10 h and then fired in air at 600 °C (heating rate: 2 °C min^−1^) for 12 h. The Li_1.5_MnTiO_4+*δ*_ nanoparticles was denoted LMTO-NP.

The bulk particles were prepared by a solid-state reaction method. Stoichiometric amounts of Li_2_CO_3_ (99.9%, Sigma-Aldrich), MnCO_3_ (99.9%, Kojundo Chemicals), and TiO_2_ (99.5%, Sigma-Aldrich) were mixed using a mortar. The resultant mixture was heated in an alumina crucible at 800 °C for 20 h at a heating rate of 2 °C min^−1^. The Li_1.5_MnTiO_4+*δ*_ bulk particles was denoted LMTO-BP.

### Structural and physical characterization

XRD data were obtained using Cu Kα radiation (Philips X’Pert) in the angle range of 10° ≤ 2*θ* ≤ 120° with a step size of 0.026°. Rietveld refinement was carried out using General Structure Analysis System (GSAS) program^23^. The sizes and morphologies of the particles were determined by FESEM and HRTEM. The FESEM images were obtained using an S-4700 (Hitachi) and TEM images were recorded using an FEI Tecnai F20 at 200 kV in the Korea Basic Science Institute (KBSI), Gwangju, Republic of Korea.

### Electrochemical characterization

The electrochemical properties of Li_1.5_MnTiO_4+*δ*_ cathodes were evaluated using Li metal as the reference electrode. For the electrochemical measurements, the electrode was fabricated by pressing a mixture of active material, conductive carbon (KETJEN black), and teflonized acetylene black (TAB) binder in a mass ratio of 75:10:15 onto a stainless steel. After that, the electrode was dried under vacuum at 120 °C for 12 h. A 2032 coin type cell, consisting of the cathode and Li metal anode separated by a polymer membrane together with glass fiber, was fabricated in an Ar-filled glove box and aged for 12 h before the electrochemical measurements. The electrolyte was a 1:1 mixture of ethylene carbonate and dimethyl carbonate containing 1 M LiPF_6_.

The charge-discharge measurements were carried out using a NAGANO BTS-2004H battery charger between 2.0 and 4.8 V *vs*. Li^+^/Li. CV measurements were taken with an Autolab electrochemical workstation at a scan rate of 0.1 mV s^−1^ between 2.0 and 4.8 V *vs*. Li^+^/Li. The EIS tests were performed using the Autolab electrochemical workstation with a voltage of 10 mV amplitude over a frequency range of 10^6^ to 10^−2^ Hz.

## Results and Discussion

The crystal structures of three samples were refined using the Rietveld method from the XRD data to explore the effect of over-stoichiometric lithium content in these cathodes. The Li_1.5_MnTiO_4+*δ*_ structure converged to a spinel phase, where Mn and Ti randomly occupy 16*d* sites and Li ions occupy 8*a* sites; this is similar to the spinel LiMn_2_O_4_ structure. The addition of excess Li led to the original spinel becoming a spinel and layered composite structure. The layered component was similar to the Li_2_Ti_1−*x*_Mn_*x*_O_3_ phase with Ti and Mn randomly occupying the 4*e* sites.

**Figure 1:**
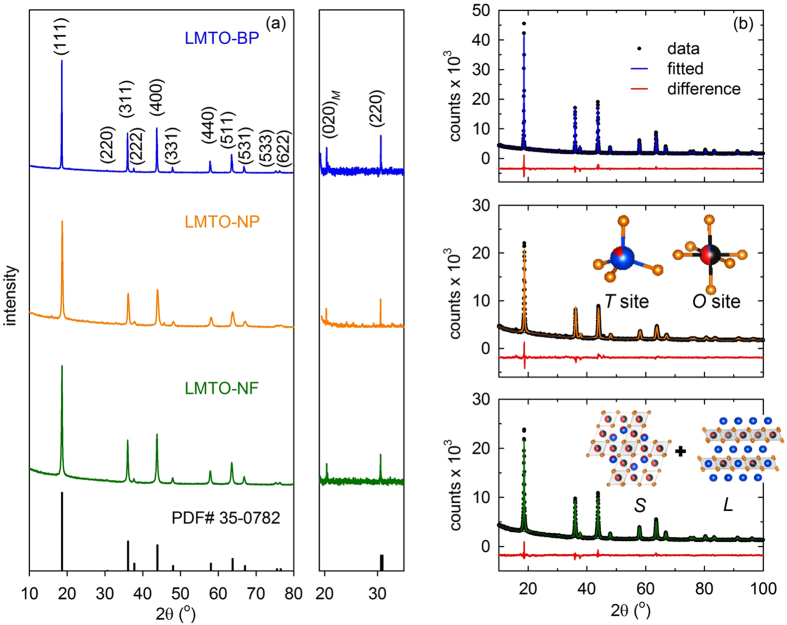
(**a**) XRD patterns of LMTO-BP, LMTO-NP, and LMTO-NF indexed against the standard LiMn_2_O_4_ (PDF No. 35-0782) phase with cubic space group *Fd*3*m*. An enlarged XRD pattern between 19–35° 2*θ* is shown demonstrating the presence of a layered superlattice peak, indicated as the (020)_*M*_ plane. (**b**) X-ray Rietveld refined patterns of LMTO-BP, LMTO-NP, and LMTO-NF with black dots representing the observed profiles, blue (LMTO-BP), orange (LMTO-NP), and green (LMTO-NF) lines representing the fitted profiles and difference profiles in red line (at the bottom); the structures of the materials were inserted include tetrahedral (*T*) sites, octahedral (*O*) sites, spinel (*S*), and layered (*L*).

Figure 1a shows the XRD pattern of the as-prepared material. All the peaks can be indexed to the cubic spinel structure in the F*d*3*m* space group; a result that agrees well with those of previous reports^8^,^24^,^25^. An extra superlattice peak, corresponding to the layered phase, is present at 20.5° in all samples. The diffraction peaks shifted toward lower diffraction angles due to the larger ionic radii of the Ti^4+^ ions that displaced the Mn^4+^ ions in the spinel sub-lattice. A (220) diffraction peak was observed for all samples, suggesting that Ti substitution induces heavy atoms, especially Mn, to occupy the Li-occupying 8*a* sites^26^,^27^,^28^,^29^,^30^. Importantly, the intensity of the (220) peak in the diffraction pattern of the Li_1.5_MnTiO_4+*δ*_ cathode was lower than that of LiMnTiO_4_ because of the excess Li^12^. Although the layered and spinel components have very similar XRD patterns, the layered component can be distinguished from the spinel structure by the unique (020)_*M*_ superlattice peaks (where *M* represents monoclinic) of the layered component, which are shown in Fig. 1a. The XRD intensities of the bulk particles were higher than the intensities of peaks of both LMTO-NP and LMTO-NF because the LMTO-BP was highly crystalline. It is worth to note that by adding excess Li, an impurity phase of TiO_2_ was undetected for all preparation methods, so it improved the purity of sample compare to report in literature^31^.

To determine phase fraction and element occupancy site, Rietveld refinement was performed. Table 1 shows the results of Rietveld refinement of the bulk particle, nanoparticle, and nanofiber samples. The refinement of each sample was performed using the spinel *Fd*3*m* structure as the major phase and the monoclinic *C*2/*c* structure as a secondary phase. The layered fraction was found to be 30% in all three samples, suggesting that the synthesis method did not affect the composite structure. However, the contents of Ti and Mn in both phases of each sample were different. For the LMTO-NF and LMTO-NP, the contents of Mn and Ti in both phases were equal while for the LMTO-BP, the spinel phase had a higher Mn content than Ti, and the layered phase had a higher Ti content than Mn. The reason for this phenomenon is that both electrospinning and sol-gel methods can control the mixing of precursors at nano-scale while for solid-state reaction method, all precursors were mixed in micro-scale. In addition, the reaction temperature also affected the contents of Ti and Mn in two phases. Moreover, as discussed earlier for all samples, Ti substitution at Mn sites in the spinel structure induces heavy Mn ions to occupy the Li 8*a* sites, and the refinement results support this for all samples. It is worth to mention that the composite Li_1.5_MnTiO_4+*δ*_ would be (1-*a*)LiMn_2−*x*_Ti_*x*_O_4_.*a*Li_2_Mn_*y*_Ti_1−*y*_O_3_^12^. For the LMTO-NP and LMTO-NF, *x* = 1 and *y* = 0.5 while for the LMTO-BP, 0 < *x* < 1 and 0 < *y* < 0.5.

**Figure 2:**
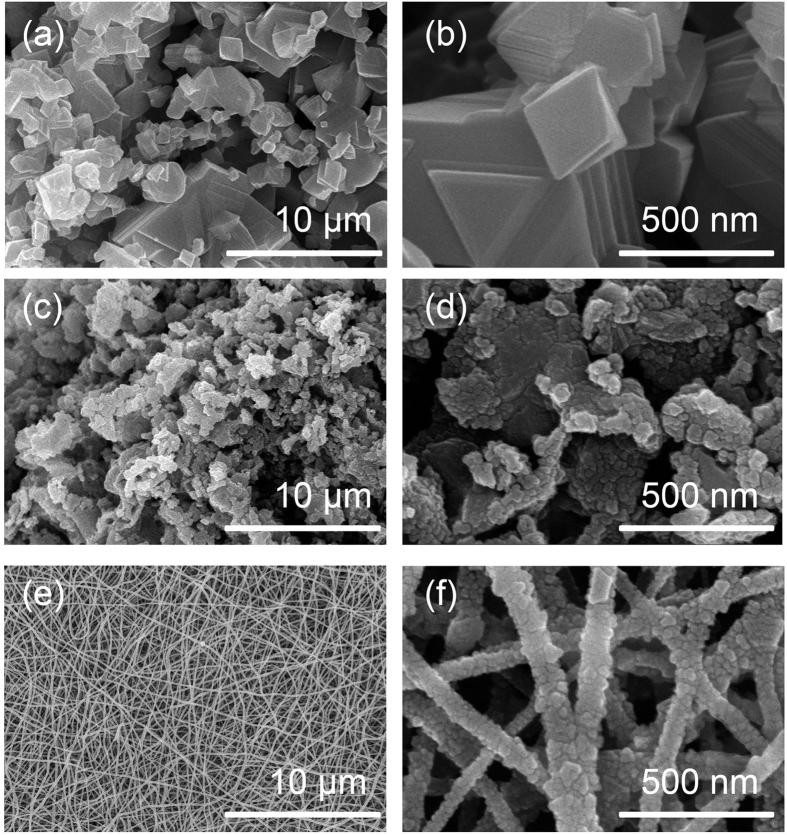
SEM images of (**a**,**b**) LMTO-BP, (**c**,**d**) LMTO-NP, and (**e**,**f**) LMTO-NF.

Figure 2 shows the SEM images of the three samples. The particle morphology is octahedral, with particle sizes of 350 nm for the LMTO-BP and about 50 nm for the LMTO-NP (Fig. 2b,d). The LMTO-NF are formed by the uniform arrangement of tiny octahedrons held together by the removable polymer network into long fibers of 30 μm length and 80 nm diameter (Fig. 2e,f). The octahedral particles in the bulk sample had different sizes, a typical feature of solid-state reactions (Fig. 2a). The nanoparticle sample showed serious particle aggregation (Fig. 2c,d), arising from the high active surface area of the nanosize particles. The average size of the particles and their morphologies strongly affect their discharge capacity and C-rate performance; in particular, the smaller the particle size is, the larger the surface area of material is. Therefore, the LMTO-NF and the LMTO-NP with sizes in the nanodomain can form good contact with the electrolyte, favoring effective Li^+^ ion exchange. LMTO-NF also prevents aggregation, which is most likely to occur in the solid-state and sol-gel samples and significantly reduces their capacities. In addition, 1-D channels of the LMTO-NF also enhance the Li diffusion kinetics. Such a structure is very favorable for Li storage because it combines efficient 1-D longitudinal electron transport, excellent connectivity between the electronic and ionic components, and shorter diffusion pathways owing to the nanoscale particle dimensions. The domain morphology of three samples was further confirmed by HRTEM and it was shown in Supplementary Figure S3.

**Figure 3:**
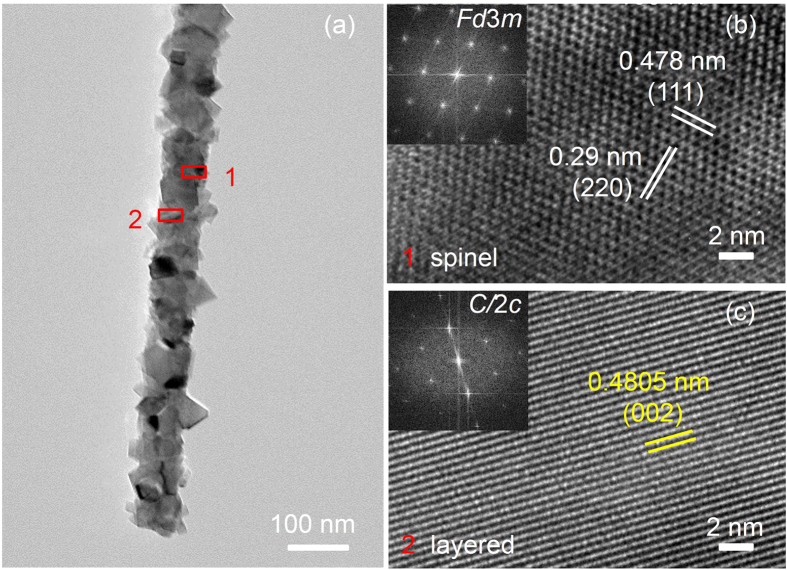
(**a**) TEM images of LMTO-NF, (**b**,**c**) enlarged HRTEM images of marked regions 1 and 2 in (**a**) with inserted fast-Fourier transform showing the spinel and layered structures with the space group indicated.

Because of possessing attractive morphology for Li^+^ diffusion with 1-D channels, the single nanofiber was more investigated. Figure 3 shows HRTEM images and Fourier image analysis that further reveal the distribution of the spinel and layered domains of the LMTO-NF. Figure 3a shows a single nanofiber, and Fig. 3c shows the typical domain structure. The LMTO-NF is formed of tiny octahedrons linked randomly together. In each octahedron, a composite spinel and layered structure was observed, and this heterostructure affects Li^+^ diffusion. The fringe spacing of 0.4805 nm corresponds to the (002) plane of Li_2_Mn_0.5_Ti_0.5_O_3_, and those of 0.478 nm and 0.29 nm correspond to the (111) plane and the (220) plane of LiMnTiO_4_, respectively. In previous reports, we have demonstrated that the spinel-layered composites show higher values of Li-ion diffusivity during both charge and discharge than that of LiMnTiO_4_ and LiMn_2_O_4_ because the layered structure of the composites improves the diffusivity of Li^+^ ions through material^12^,^32^.

**Figure 4:**
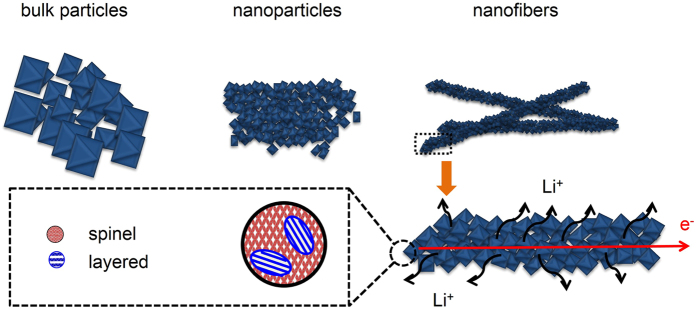
Schematic illustrations of the morphologies of LMTO-BP, LMTO-NP, and LMTO-NF. Enlarged image of marked region of a nanofiber showing Li and e diffusion. The spinel and layered structures of an octahedron are shown.

Figure 4 shows a schematic diagram for the formation of LMTO-BP, LMTO-NP, and LMTO-NF from their precursors. The solid-state reaction method does not allow for control over the size and uniformity of the particles; consequently, a broad particle size distribution was obtained and aggregation occurred. In the sol-gel method, the uniformity of particles can be controlled to the nano range, but aggregation remains a major disadvantage of this method. By utilizing the electrospinning method, LMTO-NF was formed from the tiny octahedral particles, which were wrapped together by the removable polymer network into long fibers, preventing the aggregation of the nanosize particles. 1-D nanostructures have both nanosize advantages (shorter Li-ion diffusion lengths and large surface to volume ratios for contact with the electrolyte) and facile electron transport in 1-D^33^,^34^.

XPS is a sensitive method for investigating the oxidation states at the material surface^35^. The Ti *2p* and Mn 2*p* XPS spectra of all samples are shown in Supplementary Figure S4. The Ti 2*p* of the three samples confirmed the existence of Ti^4+^ and was consistent with the reports in literature^36^. The binding energies of Ti 2*p*_3/2_ were 457.39, 457.49, and 457.57 eV for LMTO-BP, LMTO-NP, and LMTO-NF, respectively, which was smaller than that of Li_2_TiO_3_^32^,^36^. This behavior might arise from the influence of Mn and Li which is present in all samples (note that the binding energy of Mn–O is lower than that of Ti–O). The Mn 2*p*_3/2_ peaks of the composites are observed between those of MnO_2_ (642.6 eV) and Mn_2_O_3_ (641.6 eV) due to the presence of both Mn^4+^ and Mn^3+^ 36. The energy separation between the Mn 2*p*_3/2_ and Mn 2*p*_1/2_ states is 11.53 eV, which is smaller than that in LiMn_2_O_4_, arises from the Ti substitution^37^. The binding energy of Mn 2*p*_3/2_ shifted to higher energy in LMTO-NP and LMTO-NF than in LMTO-BP which suggested that there was more Mn in +4 oxidation state in LMTO-NP and LMTO-NF. It was consistent with the assumption that the LMTO-NP and LMTO-NF have higher Mn content in layered phase than the LMTO-BP.

**Figure 5:**
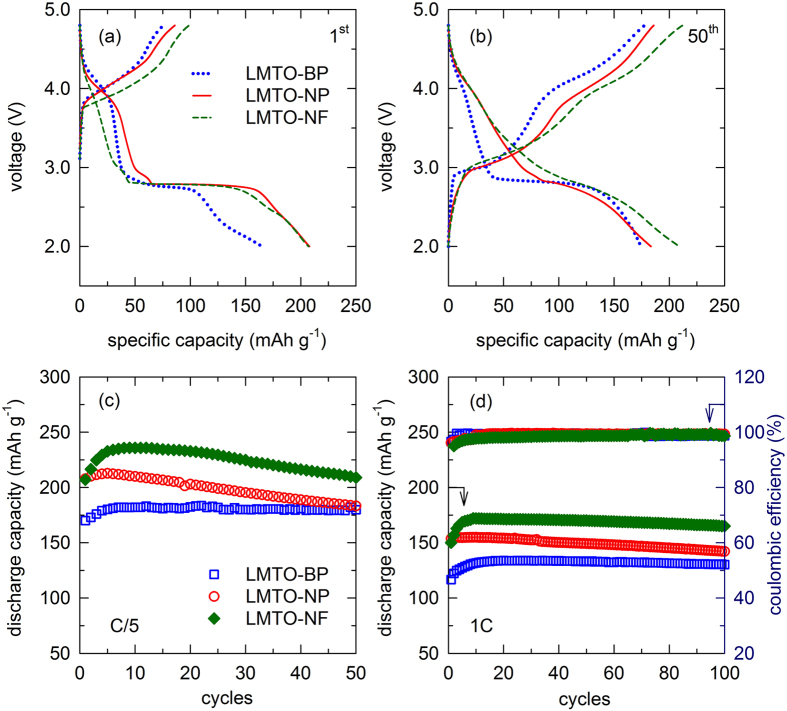
Voltage profiles of LMTO-BP, LMTO-NP, and LMTO-NF at C/5 for (**a**) the first cycle and (**b**) the 50^th^ cycle. (**c**) Cycling stability curves of LMTO-BP, LMTO-NP, and LMTO-NF samples at C/5, and (**d**) cycling stability curves of LMTO-BP, LMTO-NP, and LMTO-NF at 1 C rate.

Figure 5a,b show the charge-discharge profiles of the samples at the 1^st^ and 50^th^ cycles, respectively. In the first charging cycle, all three samples have a plateau at around 4.2 V, which is a characteristic of Li^+^ ion extraction from the tetrahedral 8*a* sites in spinel cathodes. This is followed by the oxidation of Mn^3+^ to Mn^4+^ (which was clearly observed in the CV curve)^31^. During the discharge process, voltage plateaus appear at around 4.1 and 2.8 V, corresponding to the two-phase reaction forming the LiMnTiO_4_ and Li_2_MnTiO_4_ phase where extra Li^+^ ions are inserted into the 8*a* and 16*c* sites^31^. The discharge capacities of the LMTO-NF and LMTO-NP are similar, but they are 20% higher than that of the LMTO-BP. The high surface area of both nanosize samples contributed to the increased capacity. After 50 cycles, the plateaus at 2.8 V diminished in both the LMTO-NF and LMTO-NP samples and a sloping charge/discharge profiles evolved, suggesting a gradual change in the structure of the cathode and Li^+^ storage mechanism during cycling. For the LMTO-BP, the plateau at 4.2 V, which is related to the Li^+^ ion extraction/insertion from/into the tetrahedral 8*a* sites, becomes less evident, and the plateaus at 2.8 V remain even after 50 cycles. It should be noted that the structural changes during cycling do not cause dramatic capacity fading in the LMTO-NF and LMTO-NP, as shown in Fig. 5c. This phenomenon is discussed later.

To investigate the cycling performance of these cathodes, galvanostatic charge-discharge measurements were carried out for cathodes in the voltage range of 2.0–4.8 V at room temperature. The cycling performance of the cells at C/5 and 1 C rates (current density at the 1 C was 154 mA g^−1^) are shown in Fig. 5c,d, respectively. The discharge capacities of the three samples gradually increased in the first few cycles. The capacity of the LMTO-NF increased upon cycling, reaching maxima of 235 mAh g^−1^ (726 Wh kg^−1^) at C/5 and 170 mAh g^−1^ at 1 C after eight cycles. The capacities of the LMTO-NP increased similarly, and the highest capacities obtained for this sample were 213 mAh g^−1^ (652 Wh kg^−1^) at C/5 and 155 mAh g^−1^ at 1 C after six cycles. In the bulk particle sample, the highest capacities obtained were 184 mAh g^−1^ (534 Wh kg^−1^) at C/5 and 130 mAh g^−1^ at 1 C after eight cycles. The capacity of the LMTO-NF is superior (27% higher) to that of the LMTO-BP at both high (1 C) and low C-rates (C/5). The LMTO-NP showed higher capacity than the bulk particle sample (20% for the first cycle), but after 50 cycles, the capacity was close to that of the LMTO-BP. The high capacity of the LMTO-NF can be explained by their high surface area and the lack of particle aggregation because of the large fiber length. However, at a low C-rate, the LMTO-BP showed a higher cycling stability than both the LMTO-NF and the LMTO-NP. The large surface area, which easily reacts with the electrolyte at high voltage, should be the main reason for this lower cyclability of the LMTO-NF and the LMTO-NP^38^.

However, at high C-rates, the LMTO-NF showed very high cycling stability and capacity, which arises from the robust uniform LMTO-NF composed of an ordered arrangement of tiny octahedrons without any aggregation. The cycling stability of the LMTO-NF is comparable with that of the LMTO-BP and higher than that of the LMTO-NP. Aggregation of the LMTO-NP was a significant drawback, reducing both the capacity and cycling stability compared to those of the ultralong LMTO-NF. In addition, the capacity of the LMTO-NF at 1 C rate is comparable to that of the LMTO-BP at C/5 rate, meaning that the LMTO-NF can deliver a rate five times greater than that of the LMTO-BP. The coulombic efficiencies of the three samples in the first cycle were over 100%, which is a characteristic of spinel cathodes. For the remaining cycles, the coulombic efficiencies were close to 100%, indicating negligible energy loss during charging and discharging.

**Figure 6:**
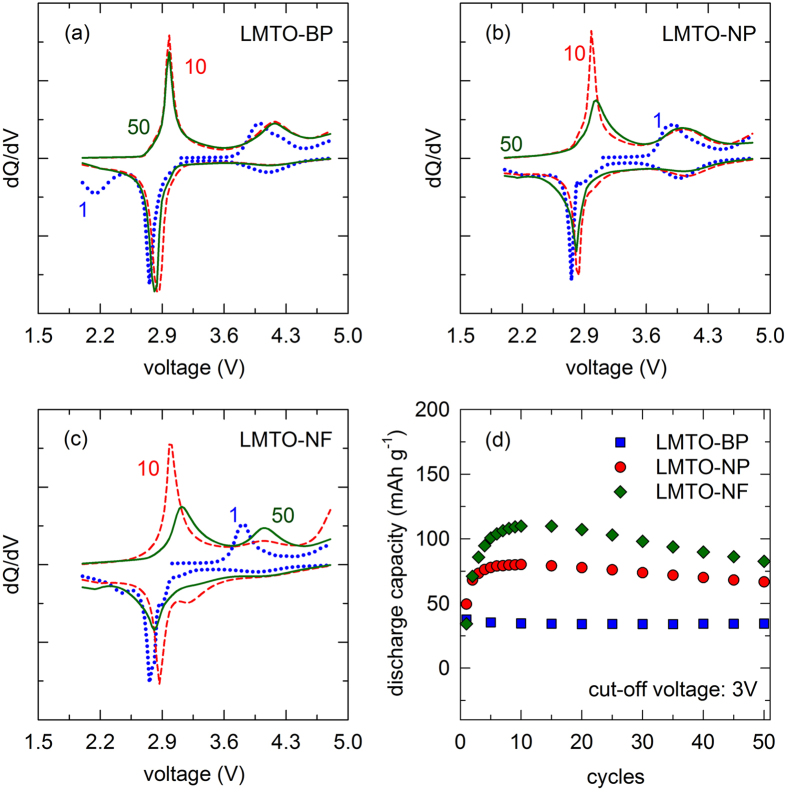
dQ/dV plot of (**a**) LMTO-BP, (**b**) LMTO-NP, and (**c**) LMTO-NF for the 1^st^, 10^th^, and 50^th^ cycle. (**d**) Cycling stability curves of LMTO-BP, LMTO-NP, and LMTO-NF sample at C/5 and cut-off voltage 3 V.

To further understand the electrochemical performance of these cathodes, dQ/dV plot of all samples at the 1^st^, 10^th^, and 50^th^ cycles are shown in Fig. 6. For the 1^st^ charge, these samples showed peak at 4 V for LMTO-BP, 3.9 V for LMTO-NP, and 3.8 V for LMTO-NF. These peaks correspond to the extraction of Li^+^ from 8*a* sites of pristine samples and the oxidation of Mn^3+^ to Mn^4+^. The high surface area of LMTO-NF and LMTO-NP are assigned for the lower oxidation peak compare to the LMTO-BP. The corresponding reduction peak of Mn^4+^/Mn^3+^ is also observed at 4.1 V for LMTO-BP and 4 V for both LMTO-NP and LMTO-NF. The typical peak at ~4.5 V and the corresponding plateau in charge-discharge profile is related to the activation of layered was not observed. However, during the 1^st^ discharge a new peak at 3 V, which stems from the reduction reaction of Mn^4+^ to Mn^3+^ in the layered MnO_2_-like component appeared for LMTO-NP and LMTO-NF while for LMTO-BP this feature was not clear^39^. Note that in layered Li_2_Mn_*y*_Ti_1−*y*_O_3,_ both Mn and Ti are +4 oxidation state and they could not be oxidized further^40^. Therefore without activation process, it was difficult for Li^+^ to reinsert into the layered structure^41^. The reason for this phenomenon is at high applied current (C/5), the peak could not be detected. At low applied current (C/10) in Supplementary Figure S5, this peak for the three samples was visible. However, the plateau in the charge profile and the corresponding peak in dQ/dV were small and therefore the activation of layered for the 1^st^ cycle was small. The strongest peak at ~2.8 V, during initial discharge of all samples is attributed to Mn^3+^/Mn^2+^. The small peak appeared less than 2.5 V for all samples is not clear at this stage. Note that this peak disappeared after 5 cycles (Supplementary Figure S5). For the subsequent cycles, the oxidation peak at 4 V region shifts to higher voltage. However, this peak of LMTO-NF and LMTO-NP still occurred at lower voltage than the one of LMTO-BP. The whole redox couple shows that the reaction of Mn^2+^/Mn^3+^ and Mn^3+^/Mn^4+^ are reversible. The reduction peak at 4 V region of LMTO-BP becomes less evident while the one of LMTO-NF and LMTO-NP was still observable and it was consistent with charge-discharge profile. The peak at 3 V region evolved and it became more evident after 10 cycles. In LMTO-NP and LMTO-NF there was a contribution of layered phase into its capacity while for LMTO-BP the contribution was less. The reason might be from the difference in particles’ size and the content of Mn in the layered phase. The LMTO-BP has a big particles’ size while the LMTO-NP and LMTO-NF have nano-size of particles so the effect of layered phase was more evident. Moreover, the activation was depended on the content of Mn in the layered; the more the Mn content in the layered phase is, the more the activation of that compound is. The LMTO-NP and LMTO-NF have higher content of Mn in layered phase than the LMTO-BP and so the activation was stronger. It was consistent with the assumption that LMTO-BP had Ti-rich layered phase while for LMTO-NF and LMTO-NP, the contents of Mn and Ti were equal. After 50 cycles, all peaks of LMTO-BP changed negligibly while all peaks of LMTO-NF and LMTO-NP were broadened and shifted to a lower voltage (which was clearly observed in CV curves in Supplementary Figure S6). It suggests that the layered gradually change to spinel phase. To evaluate the contribution of layered phase on overall capacity of electrode, the capacity of all electrodes versus cycles with 3 V cut-off voltage were plotted in Fig. 6d. As we can see from Fig. 6d, the contribution of high capacity region (over 3 V) for LMTO-BP was unchanged during cycling while the one for LMTO-NF and LMTO-NP was increasing for the first 10 cycles and then decreasing. The loss of capacity over 3 V region caused the overall capacity fading. The contribution of over 3 V capacity was 20% of overall capacity. For LMTO-NP and LMTO-NF, the contribution of over 3 V region was increasing and reached 39% for LMTO-NP and 45% for LMTO-NF. From the above discussion, the increase in the capacity in the first few cycles could be explained by the activation of layered phase. The activation was not complete in 1^st^ cycle and it induced the structural change from layered to spinel-like, by the diffusion of transition metal ions into Li layers, which was consistent with the report of Ye *et al*.^42^.

To check the structure of all samples after cycling, three cells were opened and measured *ex situ* XRD. As we can see from Supplementary Figure S7, there was no evident for the cubic to tetragonal phase transformation during electrochemical cycling. The result showed that high concentration of Ti substitution can suppress the Jahn-Teller distortion, resulting in improved structural integrity which can improve the cycling performance and it was consistent with reports in the references. During cycling, the layered structure gradually transformed to a spinel structure, leading to a decrease in the discharge capacity. The (020)_*M*_ peak could not be detected after 50 cycles. However, the structure change did not decrease capacity of LMTO-BP (capacity retention is 98%). The structure change affected the discharge capacity of LMTO-NP and LMTO-NF. It was reasonable because the layered phase contributed to capacity of LMTO-NP and LMTO-NF while it did not contribute much to LMTO-BP.

**Figure 7:**
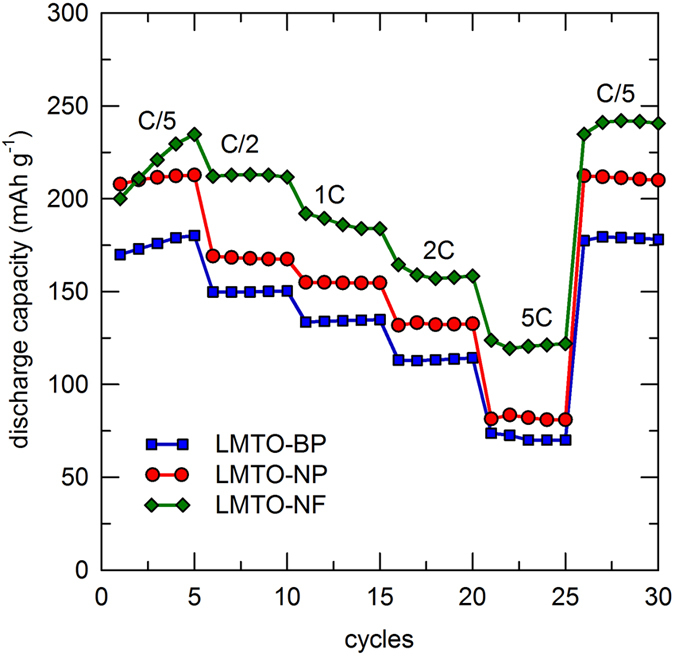
Cycling stability curves of LMTO-BP, LMTO-NP, and LMTO-NF at different C-rates.

The performance of these materials at different C-rates is shown in Fig. 7. The rate capabilities of the LMTO-NF were significantly higher than those of the LMTO-NP and the LMTO-BP. At C/5 rate, all three samples showed increased discharge capacities in the first few cycles due to the activation of the electrode material. The LMTO-NF showed a dramatic increase in capacity, while the LMTO-NP and the LMTO-BP exhibited a marginal increase in the capacity. This phenomenon in the nanofiber sample is attributed to the high surface area, 1-D diffusion pathways, and excellent contact between the cathode and electrolyte where the redox reaction occurs. The LMTO-NP also had a high surface area, but aggregation reduced contact with the electrolyte. In addition, the aggregation of LMTO-NP disfavors Li^+^ diffusion and electron transport because of the increase in grain boundaries, which act as barriers^21^, and these factors affected both the capacity and C-rate performance. According to the reports in literature, some Mn^3+^ ions in the lithium-rich composites could improve the electronic conductivity and furthermore the electrochemical performance^39^. The nanofiber cathode delivered stable capacities of 219 mAh g^−1^ at C/5, 212 mAh g^−1^ at C/2, 187 mAh g^−1^ at 1 C, 159 mAh g^−1^ at 2 C, and 121 mAh g^−1^ at 5 C. The nanofiber sample delivered 70% greater capacity than that of the bulk particle sample at 5 C. As shown by the results of the C-rate experiments, the unique, ultralong morphology of the LMTO-NF enabled high capacities at rates five times higher than those of the LMTO-BP. More importantly, the reversible capacity of the cathode was 100% at C/5 after a full sequence measurement with high currents of up to 5 C.

**Figure 8:**
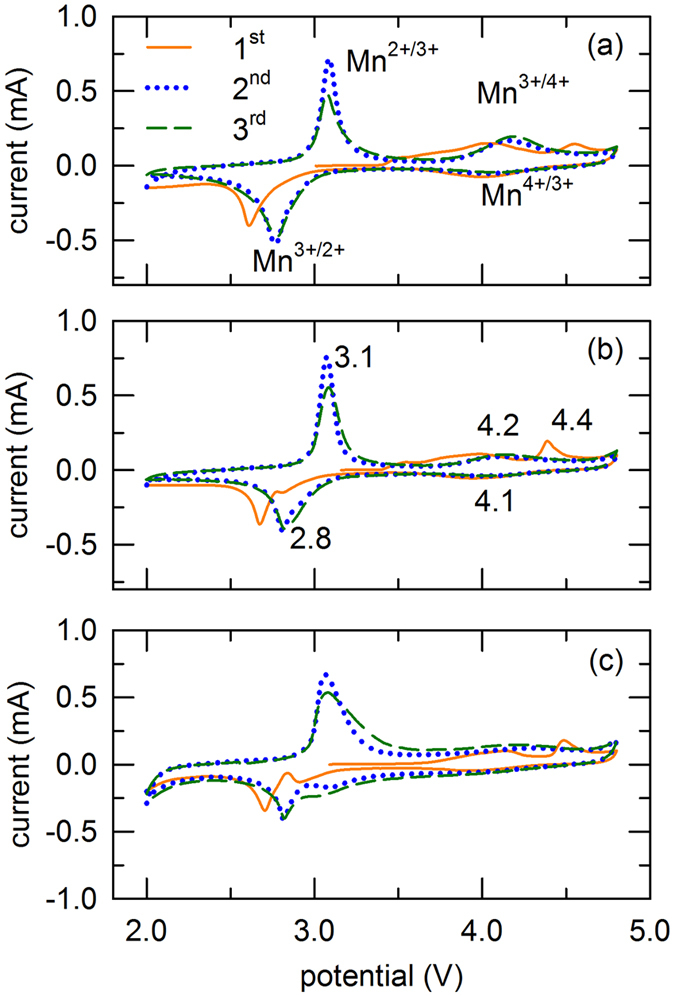
CV curves of (**a**) LMTO-BP, (**b**) LMTO-NP, and (**c**) LMTO-NF in the potential window of 2.0–4.8 V at a scan rate of 0.05 mVs^−1^.

CV was used to understand the redox activity of the samples in the voltage range of 2.0–4.8 V and a scan rate of 0.05 mV s^−1^, and the CV curves are shown in Fig. 8. In the first anodic segment of all samples, the peak at ~4.0 V is attributed to the oxidation of Mn^3+^, and the peak at ~4.4 V is associated with oxygen evolution and Li^+^ extraction from its layered component to form MnO_2_-like component^43^. The peaks of LMTO-NP and LMTO-NF shifted to lower potential and showed stronger compare to LMTO-BP caused by higher reaction area and higher Mn content in layered phase. It was consistent with charge-discharge profiles and dQ/dV plot (Supplementary Figure S5). For the first cathodic segment, peaks at ~3 V of LMTO-NP and LMTO-NF corresponded to the reduction of Mn^4+^ in layered phase while peaks at 2.7 V corresponded to the reduction of Mn^3+^ in spinel phase. The peak at 3 V in LMTO-BP was not clear because the contribution of layered phase into its capacity was less. For the subsequent cycles, two types of oxidation/reduction couples at 3.1/2.8 V and 4.2/4.1 V in all three samples, corresponding to the Mn^2+^/Mn^3+^ and Mn^3+^/Mn^4+^ redox reactions, respectively^31^ were shifted to higher potential. These peaks are consistent with the charge-discharge profiles (Fig. 5a,b). This behavior is characteristic of the spinel Li_*x*_MnTiO_4_ phase, in which Li is inserted into tetrahedral sites at potentials greater than 4.0 V, corresponding to 0 < *x* ≤ 1, and into the octahedral sites over the 3.0 V, corresponding to 1 < *x* ≤ 2^44^. According to previous reports, Ti^4+^ is electrochemically inactive in this voltage window, so these redox reactions are related to Mn-based species, namely Mn^2+^/Mn^3+^ and Mn^3+^/Mn^4+^ 8,31,45. The intensities of the peaks and the integrated areas also demonstrate the capacity of the material. The peak intensity in the high voltage range was weak, which indicates that the contribution of the Mn^3+^/Mn^4+^ redox reaction to the capacity of the materials was lower than that of the Mn^2+^/Mn^3+^ redox reaction for all composites.

**Figure 9:**
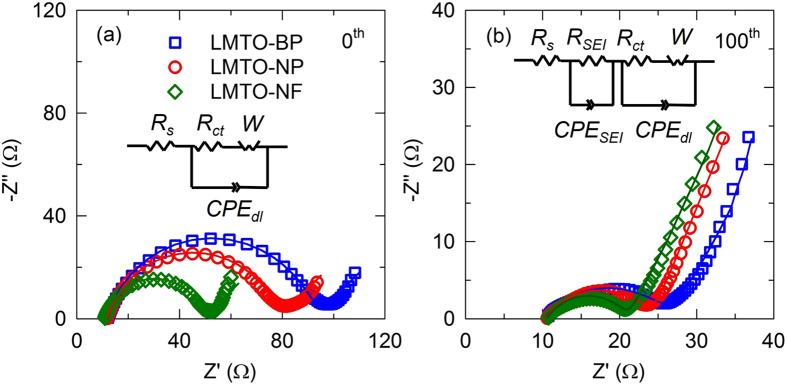
Electrochemical impedance spectra of LMTO-BP, LMTO-NP, and LMTO-NF (**a**) before and (**b**) after 100 cycles at 1C with the equivalent circuits used to model the EIS.

Figure 9a,b show the Nyquist plots of the LMTO-NF, LMTO-NP, and LMTO-BP before and after 100 cycles at 1C, respectively. Before cycling, the Nyquist plots showed only a single semicircle. An equivalent circuit was used to simulate the impedance spectra, as shown in the inset of Fig. 8a,b. In the equivalent circuits, *R*_*s*_, *R*_*ct*_ and *R*_*SEI*_ are the solution resistance, charge-transfer resistance, and solid-electrolyte interphase (SEI) resistance, respectively. *CPE*_*dl*_ and *CPE*_*SEI*_ are constant phase elements of the double layer and SEI. The simulated results were shown in Table S1. A sloped line appears in the low-frequency region, which is attributed to Li^+^ diffusion (Warburg diffusion) in the electrode bulk. The *R*_*ct*_ of the LMTO-NF (38 Ω) is much lower than that of the LMTO-NP (68 Ω) and LMTO-BP (81 Ω) due to the higher surface area of the LMTO-NF and lack of aggregation, which improved the contact between the electrode and electrolyte compared to that in the LMTO-NP and LMTO-BP. After 100 cycles, all samples were rested for 2 h before measuring the EIS spectra. The Nyquist plots after 100 cycles showed two semicircles in the high-to-medium frequency region. The first semicircle in the high-frequency region is associated with resistance due to Li^+^ ion diffusion in the surface layer, including the SEI layer^43^. The second semicircle in the medium frequency region is related to the charge-transfer resistance between the surface layer and the active cathode mass^46^. The overall resistance of the LMTO-NF was the lowest of the three samples, which led to the enhanced electrochemical cycling performance of the LMTO-NF.

## Conclusions

The promising spinel-layered Li_1.5_MnTiO_4+*δ*_ cathode materials with different morphology were synthesized and investigated. The LMTO-BP with Mn-rich spinel phase and Ti-rich layered phase showed excellent cycle stability (98% capacity retention after 100 cycles at 1C). The LMTO-NP and the LMTO-NF have higher content of Mn in layered phase compare to the bulk particle, which affected strongly to electrochemical performance of them. It contributed to capacity of these cathodes over 3 V region and increasing in the first few cycles. The LMTO-NF has excellent performance, delivering a high capacity at a rate five times higher than that of the LMTO-BP. An enhanced capacity retention of 94% was achieved at 1 C rate even after 100 cycles. By possessing outstanding properties, we believe that this material is a potential cathode for the development of rechargeable Li-ion batteries.

## Additional Information

**How to cite this article:** Hung Vu, N. *et al*. High-performance spinel-rich Li_1.5_MnTiO_4+*d*_ ultralong nanofibers as cathode materials for Li-ion batteries. *Sci. Rep.* **7**, 45579; doi: 10.1038/srep45579 (2017).

**Publisher's note:** Springer Nature remains neutral with regard to jurisdictional claims in published maps and institutional affiliations.

## Supplementary Material

Supplementary Information

## Figures and Tables

**Table 1:** Rietveld refinement and crystal data obtained from the XRD data.

formula	LMTO-BP	LMTO-NP	LMTO-NF
space group	*Fd*3*m*	*Fd*3*m*	*Fd*3*m*
phase fraction	70%	70%	70%
a = b = c (Å)	8.2794 (5)	8.2712 (11)	8.2792 (5)
8*a* site	92.5% Li	92.5% Li	92.5% Li
occupancy	7.5% Mn	7.5% Mn	7.5% Mn
16*d* site	2.5% Li	2.5% Li	2.5% Li
occupancy	52.5% Mn	47.5% Mn	47.5% Mn
	45% Ti	50% Ti	50% Ti
space group	*C*2/*c*	*C*2/*c*	*C*2/*c*
phase fraction	30%	30%	30%
*a* (Å)	5.0491 (24)	5.0431 (33)	5.1815 (28)
*b* (Å)	8.7153 (41)	8.7303 (48)	8.5608 (39)
*c* (Å)	9.7815 (20)	9.7954 (28)	9.7380 (22)
*β* (°)	100.1744 (33)	100.333 (23)	102.2491 (31)
4*e* site	32.5% Mn	50% Mn	50% Mn
occupancy	67.5% Ti	50% Ti	50% Ti
*R*_*p*_ (%)	2.59	2.67	2.75
*R*_*wp*_ (%)	3.66	3.75	3.74
*χ*^2^ (%)	2.95	3.327	2.735

The numbers in parentheses are the estimated standard deviations of the last significant figure.

## References

[b1] Ma J., Hu P., Cui G. & Chen L. Surface and Interface Issues in Spinel LiNi_0.5_Mn_1.5_O_4_: Insights into a Potential Cathode Material for High Energy Density Lithium Ion Batteries, Chem. Mater. 28, 3578–3606 (2016).

[b2] Lee H.-W. . Ultrathin Spinel LiMn_2_O_4_ Nanowires as High Power Cathode Materials for Li-Ion Batteries. Nano Lett. 10, 3852–3856 (2010). 20795626 10.1021/nl101047f

[b3] Jafta C. J., Mathe M. K., Manyala N., Roos W. D. & Ozoemena K. I. Microwave-Assisted Synthesis of High-Voltage Nanostructured LiMn_1.5_Ni_0.5_O_4_ Spinel: Tuning the Mn^3+^ Content and Electrochemical Performance. ACS. Appl. Mater. Interfaces 5, 7592–7598 (2013). 23855720 10.1021/am401894t

[b4] Thackeray M. M. Manganese oxides for lithium batteries. Prog. Solid State Chem. 25, 1–71 (1997).

[b5] Goodenough J. B. & Park K.-S. The Li-Ion Rechargeable Battery: A Perspective. J. Am. Chem. Soc. 135, 1167–1176 (2013). 23294028 10.1021/ja3091438

[b6] Hernán L., Morales J., Sánchez L. & Santos J. Use of Li–*M*–Mn–O [*M* = Co, Cr, Ti] spinels prepared by a sol-gel method as cathodes in high-voltage lithium batteries. Solid State Ion. 118, 179–185 (1999).

[b7] Krins N. . LiMn_2−*x*_Ti_*x*_O_4_ spinel-type compounds (x ≤ 1): Structural, electrical and magnetic properties. Solid State Ion. 177, 1033–1040 (2006).

[b8] Wang S. . Toward high capacity and stable manganese-spinel electrode materials: A case study of Ti-substituted system. J. Power Sources 245, 570–578 (2014).

[b9] Qian Y. . Investigation of the Effect of Extra Lithium Addition and Postannealing on the Electrochemical Performance of High-Voltage Spinel LiNi_0.5_Mn_1.5_O_4_ Cathode Material. J. Phys. Chem. C. 118, 15581–15589 (2014).

[b10] Arunkumar P., Jeong W. J., Won S. & Im W. B. Improved electrochemical reversibility of over-lithiated layered Li_2_RuO_3_ cathodes: Understanding aliovalent Co^3+^ substitution with excess lithium. J. Power Sources 324, 428–438 (2016).

[b11] Ma D., Zhang P., Li Y. & Ren X. Li_1.2_Mn_0.54_Ni_0.13_Co_0.13_O_2_-Encapsulated Carbon Nanofiber Network Cathodes with Improved Stability and Rate Capability for Li-ion Batteries. Scientific Reports 5, 11257 (2015). 26053003 10.1038/srep11257 PMC4459222

[b12] Vu N. H. . Effects of excess Li on the structure and electrochemical performance of Li_1+*z*_MnTiO_4+*δ*_ cathode for Li-ion batteries, Electrochim. Acta 225, 458–466 (2017).

[b13] Pang S. . Electrostatic assembly of mesoporous Li_4_Ti_5_O_12_/graphene hybrid as high-rate anode materials, Scr. Mater. 69, 171–174 (2013).

[b14] Liu Z., Chai J., Xu G., Wang Q. & Cui G. Functional lithium borate salts and their potential application in high performance lithium batteries, Coord. Chem. Rev. 292, 56–73 (2015).

[b15] Zhao Y. . Nitridated mesoporous Li_4_Ti_5_O_12_ spheres for high-rate lithium-ion batteries anode material, J. Solid State Electrochem. 17, 1479–1485 (2013).

[b16] Zhou H. . Preparation and Characterization of Ultralong Spinel Lithium Manganese Oxide Nanofiber Cathode via Electrospinning Method, Electrochim. Acta 152, 274–279 (2015).

[b17] Zhou H. . Fabrication and electrochemical characteristics of electrospun LiMn_2_O_4_ nanofiber cathode for Li-ion batteries, Mater. Lett. 117, 175–178 (2014).

[b18] Zhang C.-L. & Yu S.-H. Nanoparticles meet electrospinning: recent advances and future prospects. Chem. Soc. Rev. 43, 4423–4448 (2014). 24695773 10.1039/c3cs60426h

[b19] Anjusree G. S. . Fabricating fiber, rice and leaf-shaped TiO_2_ by tuning the chemistry between TiO_2_ and the polymer during electrospinning. RSC Adv. 3, 16720–16727 (2013).

[b20] Min J. W., Yim C. J. & Im W. B. Facile Synthesis of Electrospun Li_1.2_Ni_0.17_Co_0.17_Mn_0.5_O_2_ Nanofiber and Its Enhanced High-Rate Performance for Lithium-Ion Battery Applications. ACS. Appl. Mater. Interfaces 5, 7765–7769 (2013). 23905782 10.1021/am402484f

[b21] Mai L. . Electrospun Ultralong Hierarchical Vanadium Oxide Nanowires with High Performance for Lithium Ion Batteries. Nano Lett. 10, 4750–4755 (2010). 20954742 10.1021/nl103343w

[b22] Wang H., Ma D., Huang X., Huang Y. & Zhang X. General and Controllable Synthesis Strategy of Metal Oxide/TiO_2_ Hierarchical Heterostructures with Improved Lithium-Ion Battery Performance. Scientific Reports 2, 701 (2012). 23050085 10.1038/srep00701 PMC3463005

[b23] Larson A. C. & Von Dreele R. B. GSAS, Los Alamos National Laboratory Report LAUR (1994).

[b24] Arillo M. A. . Structural characterisation and physical properties of Li*M*MnO_4_ (*M* = Cr, Ti) spinels, Solid State Sci. 7, 25–32 (2005).

[b25] Arillo M. Á., López M. L., Pico C. & Veiga M. L. Structural, thermal and magnetic properties of LiMnTiO_4_ spinel in different atmospheres. Solid State Sci. 10, 1612–1619 (2008).

[b26] Song D., Ikuta H., Uchida T. & Wakihara M. The spinel phases LiAl_*y*_Mn_2−*y*_O_4_ (*y* = 0, 1/12, 1/9, 1/6, 1/3) and Li(Al,*M*)_1/6_Mn_11/6_O_4_ (*M* = Cr, Co) as the cathode for rechargeable lithium batteries. Solid State Ion. 117, 151–156 (1999).

[b27] Lee Y.-S. & Yoshio M., Unique Aluminum Effect of LiAl_*x*_Mn_2−*x*_O _4_ Material in the 3 V Region, Electrochem. Solid-State Lett. 4, A85–A88 (2001).

[b28] Fey G. T.-K., Lu C.-Z. & Kumar T. P. Preparation and electrochemical properties of high-voltage cathode materials, Li*M*_*y*_Ni_0.5*−y*_Mn_1.5_O_4_ (*M* = Fe, Cu, Al, Mg; *y* = 0.0–0.4). J. Power Sources 115, 332–345 (2003).

[b29] Xiao L. . Enhanced electrochemical stability of Al-doped LiMn_2_O_4_ synthesized by a polymer-pyrolysis method, Electrochim. Acta 54, 545–550 (2008).

[b30] Xiong L., Xu Y., Tao T. & Goodenough J. B. Synthesis and electrochemical characterization of multi-cations doped spinel LiMn_2_O_4_ used for lithium ion batteries, J. Power Sources 199, 214–219 (2012).

[b31] Chen R. . Reversible Li^+^ Storage in a LiMnTiO_4_ Spinel and Its Structural Transition Mechanisms. J. Phys. Chem. C. 118, 12608–12616 (2014).

[b32] Lu J. . Nanoscale Coating of Li*M*O_2_ (*M* = Ni, Co, Mn) Nanobelts with Li^+^-Conductive Li_2_TiO_3_: Toward Better Rate Capabilities for Li-Ion Batteries. J. Am. Chem. Soc. 135, 1649–1652 (2013). 23301844 10.1021/ja308717z

[b33] Liu L. . Electrospun porous lithium manganese phosphate-carbon nanofibers as a cathode material for lithium ion batteries. J. Mater. Chem. A. 3, 17713–17720 (2015).

[b34] Liu B. . Encapsulation of MnO Nanocrystals in Electrospun Carbon Nanofibers as High-Performance Anode Materials for Lithium-Ion Batteries. Scientific Reports 4, 4229 (2014). 24598639 10.1038/srep04229 PMC3944319

[b35] Biesinger M. C. . Resolving surface chemical states in XPS analysis of first row transition metals, oxides and hydroxides: Cr, Mn, Fe, Co and Ni. Appl. Surf. Sci. 257, 2717–2730 (2011).

[b36] Briggs D. Handbook of X-ray Photoelectron Spectroscopy C. D. Wanger, W. M. Riggs, L. E. Davis, J. F. Moulder and G. E. Muilenberg Perkin-Elmer Corp., Physical Electronics Division, Eden Prairie, Minnesota, USA, 1979. 190 pp., Surf. Interface Anal. 3 (1981).

[b37] Ramana C. V., Massot M. & Julien C. M. XPS and Raman spectroscopic characterization of LiMn_2_O_4_ spinels. Surf. Interface Anal. 37, 412–416 (2005).

[b38] Okubo M. . Nanosize Effect on High-Rate Li-Ion Intercalation in LiCoO_2_ Electrode, J. Am. Chem. Soc. 129, 7444–7452 (2007). 17511453 10.1021/ja0681927

[b39] Fu C., Li G., Luo D., Zheng J. & Li L. Gel-combustion synthesis of Li_1.2_Mn_0.4_Co_0.4_O_2_ composites with a high capacity and superior rate capability for lithium-ion batteries. J. Mater. Chem. A 2, 1471–1483 (2014).

[b40] Robertson A. D. & Bruce P. G. Mechanism of Electrochemical Activity in Li_2_MnO_3_, Chem. Mater. 15, 1984–1992 (2003).

[b41] Lu Z. & Dahn J. R. Understanding the Anomalous Capacity of Li /Li[Ni_*x*_Li_(1/3−2*x*/3)_Mn_(2/3−*x*/3)_]O_2_ Cells Using *In Situ* X-Ray Diffraction and Electrochemical Studies. J. Electrochem. Soc. 149, A815–A822 (2002).

[b42] Ye D. . Understanding the Origin of Li_2_MnO_3_ Activation in Li-Rich Cathode Materials for Lithium-Ion Batteries. Adv. Funct. Mater. 25, 7488–7496 (2015).

[b43] Thackeray M. M., Johnson C. S., Vaughey J. T., Li N. & Hackney S. Advances in Manganese-oxide ‘Composite’ Electrodes for Lithium-Ion Batteries, J. Mater. Chem. 15, 2257–2267 (2005).

[b44] Jang Y. I., Huang B., Wang H., Sadoway D. R. & Chiang Y. M. Electrochemical Cycling‐Induced Spinel Formation in High‐Charge‐Capacity Orthorhombic LiMnO_2_, J. Electrochem. Soc. 146, 3217–3223 (1999).

[b45] Kalathil A. K., Arunkumar P., Kim D. H., Lee J.-W. & Im W. B. Influence of Ti^4+^ on the Electrochemical Performance of Li-Rich Layered Oxides - High Power and Long Cycle Life of Li_2_Ru_1−*x*_Ti_*x*_O_3_ Cathodes. ACS. Appl. Mater. Interfaces 7, 7118–7128 (2015). 25762101 10.1021/am507951x

[b46] Lin J. . Li-rich layered composite Li[Li_0.2_Ni_0.2_Mn_0.6_]O_2_ synthesized by a novel approach as cathode material for lithium ion battery. J. Power Sources 230, 76–80 (2013).

