# Supplementary material for: High-performance spinel-rich Li1.5MnTiO4+δ ultralong nanofibers as cathode materials for Li-ion batteries
Source: Sci Rep. 2017 Mar 31;7:45579. doi: 10.1038/srep45579 (PMC5374536; doi:10.1038/srep45579)
Supplement: Supplementary Information [file srep45579-s1.pdf]

High-performance spinel-rich $Li_{1.5}MnTiO_{4+\delta}$ ultralong nanofibers as cathode materials for Li-ion batteries

Ngoc Hung Vu, Paulraj Arunkumar, and Won Bin Im*

School of Materials Science and Engineering and Optoelectronics Convergence Research Center, Chonnam National University, 77 Yongbong-ro, Buk-gu, Gwangju 61186, Republic of Korea.

*****To whom correspondence should be addressed

Tel : +82-62-530-1715

Fax : +82-62-530-1699

E-mail: imwonbin@jnu.ac.kr

Table S1. The simulated results from electrochemical impedance spectra of all samples before and after 100 cycles at 1C.

sample	before cycling		after 100 cycles		
	R_s (Ω)	R_{ct} (Ω)	R_s (Ω)	R_{ct} (Ω)	R_{SEI} (Ω)
LMTO-BP	12	81	11.6	436	16.8
LMTO-NP	11.8	68	10.3	240	14
LMTO-NF	10.9	38	7.2	145	10.6

Figures

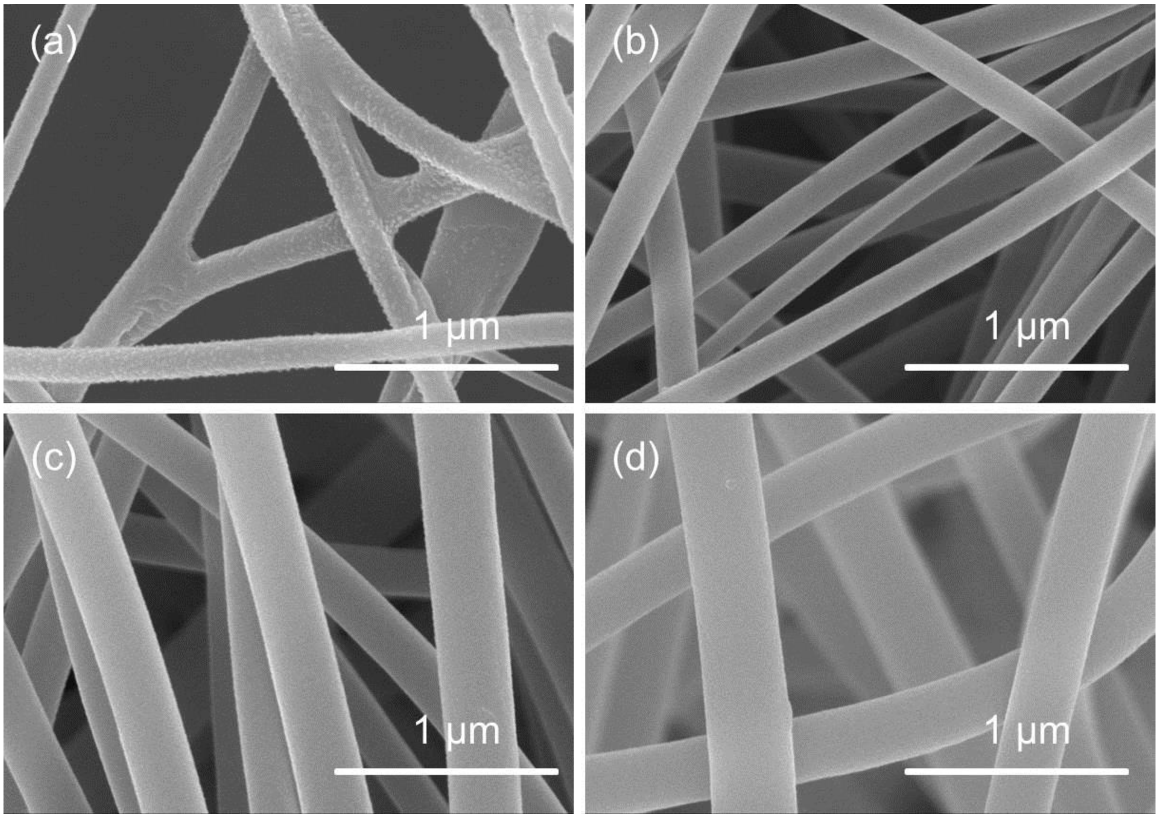

Figure S1. SEM of as-spun nanofibers sample with different PVP concentration: (a) 0.05 g/ml, (b) 0.1 g/ml, (c) 0.15 g/ml, and (d) 0.2 g/ml.

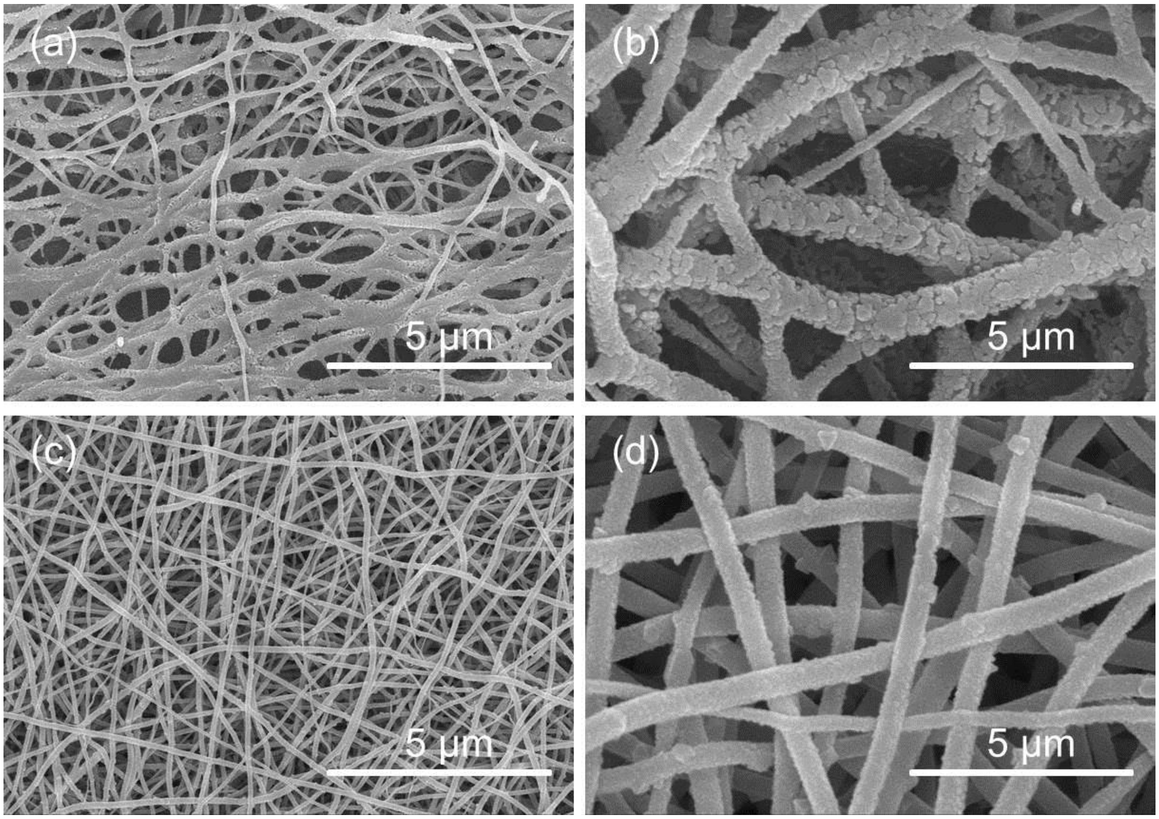

Figure S2. SEM images of nanofibers after annealing using (a, b) PVA, and (c, d) PVP as a template.

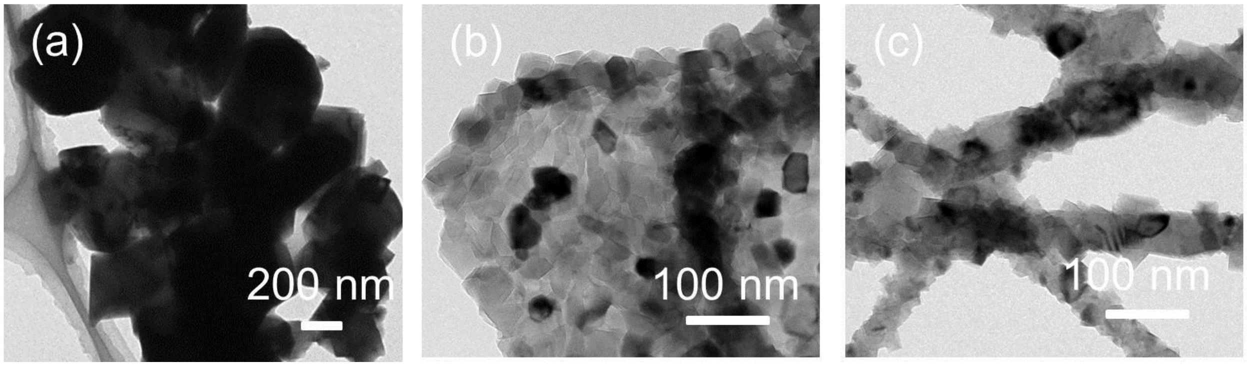

Figure S3. HRTEM images of (a) LMTO-BP, (b) LMTO-NP, and (c) LMTO-NF.

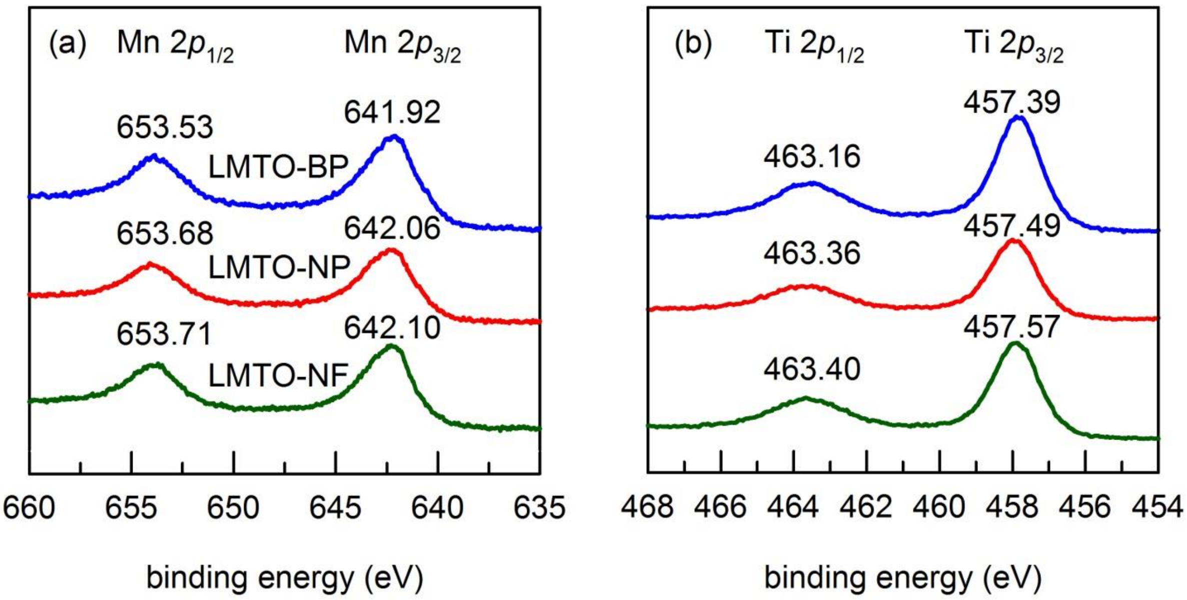

Figure S4. XPS spectra of (a) Mn 2*p* and (b) Ti 2*p* of LMTO-BP, LMTO-NP, and LMTO-NF.

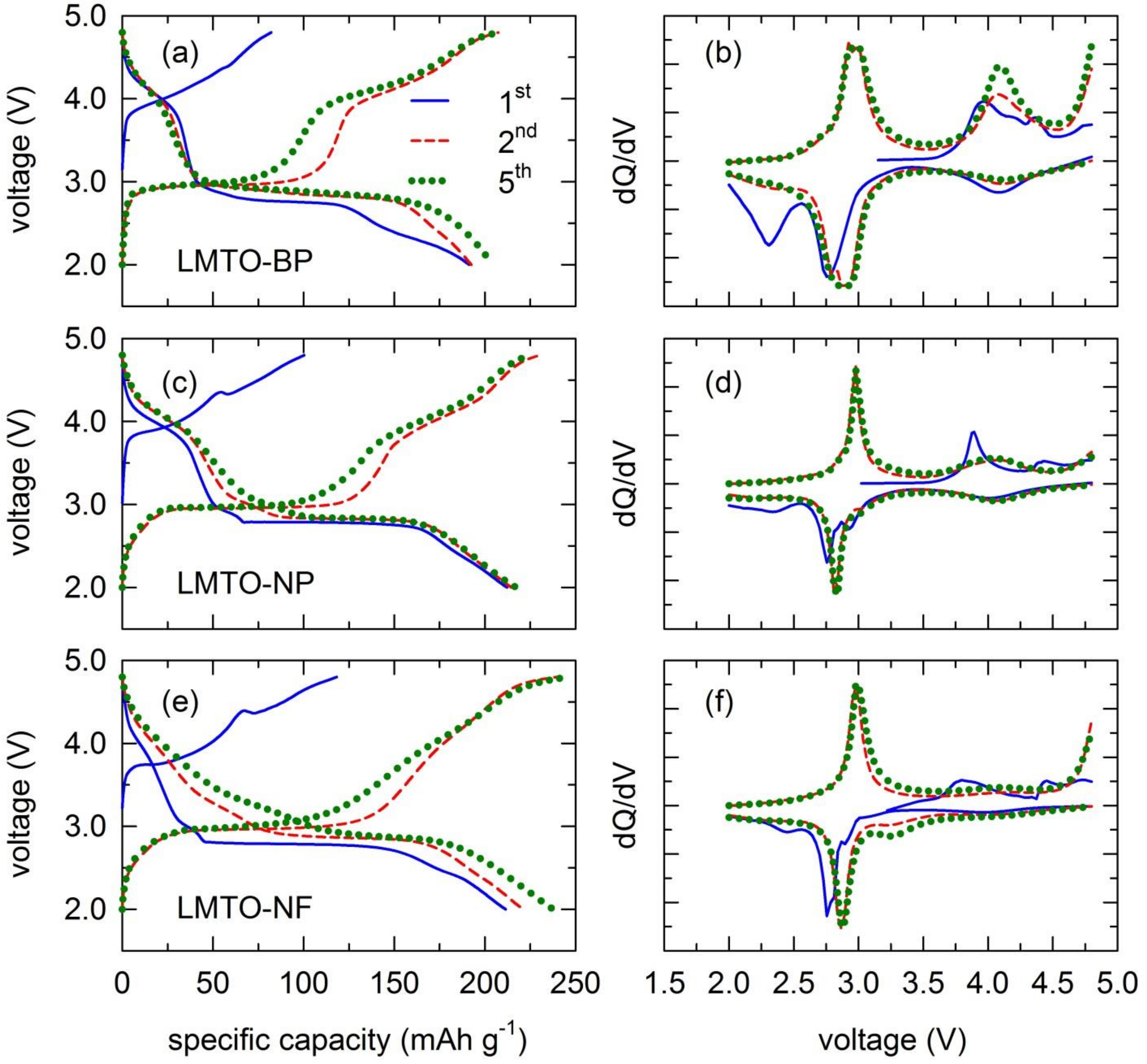

Figure S5. Voltage profile and dQ/dV plot of (a, b) LMTO-BP, (c, d) LMTO-NP, and (e, f) LMTO-NF at C/10 for the 1st, 2nd, and 5th cycle.

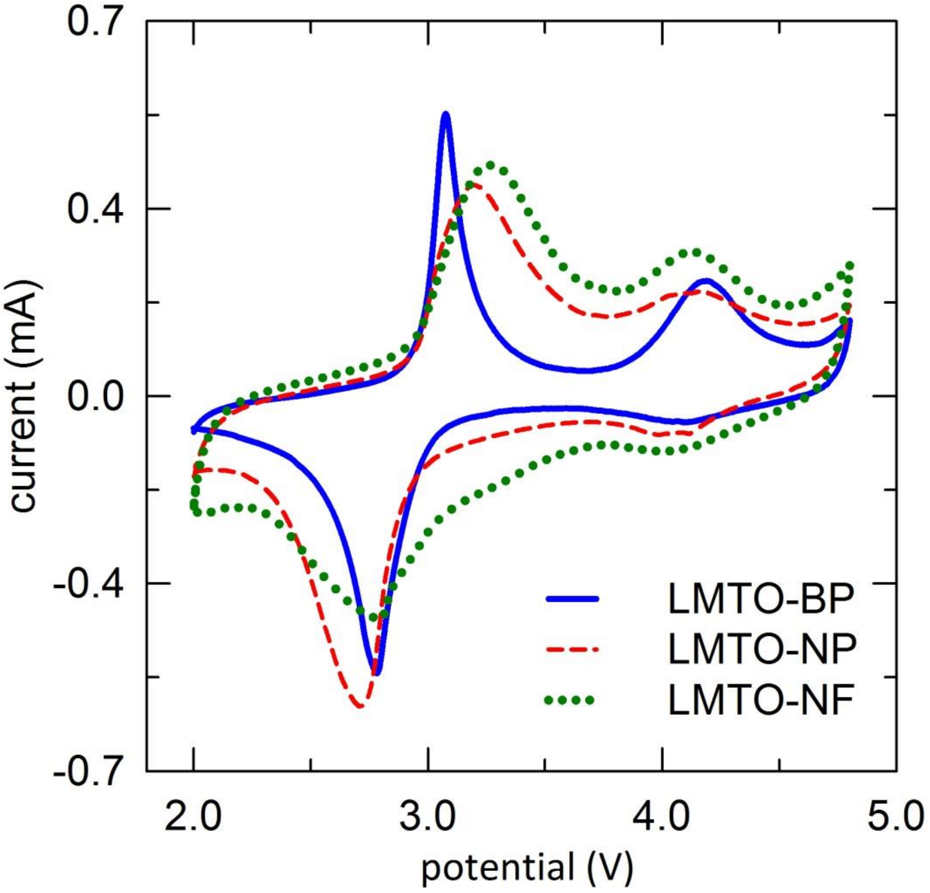

Figure S6. CV curves of (a) LMTO-BP, (b) LMTO-NP, and (c) LMTO-NF after 50 cycles, at C/5 in the potential window of 2.0–4.8 V at a scan rate of 0.05 mVs^{-1}.

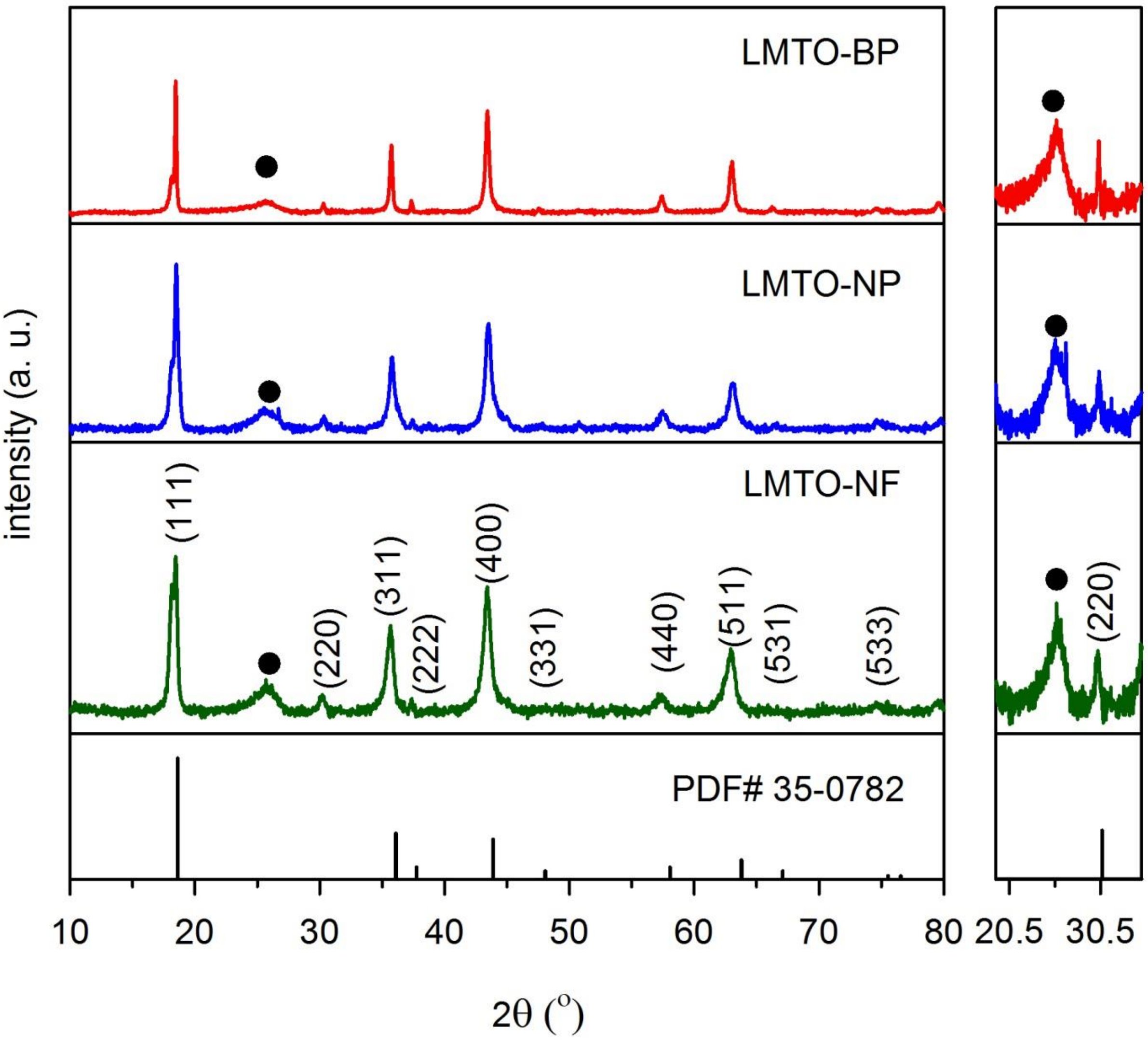

Figure S7. *Ex situ* XRD of LMTO-BP, LMTO-NP and LMTO-NF sample after 50 cycles, at C/5. The black circles represents the presence of carbon in the electrode. An enlarged XRD pattern in the Bragg's angle range of 19–35° was shown to depict the absence of a layered superlattice peak denoted by the $(020)_M$ plane.
